# Peer Mentoring as a Community of Practice in Medical Education

**DOI:** 10.1111/tct.70238

**Published:** 2025-11-05

**Authors:** Jun Jie Lim, Vivian Andaya Verbo, Gunjan Khandelwal, Nadine Hayudini Nograles

**Affiliations:** ^1^ Newcastle University Medicine Malaysia, Faculty of Medical Sciences Newcastle University Iskandar Puteri Malaysia; ^2^ School of Medicine Newcastle University Newcastle upon Tyne UK

**Keywords:** Community of Practice (CoP), medical education, mentorship programmes, near‐peer mentoring, peer mentoring, student development

## Abstract

**Background:**

First‐year medical students often face significant challenges adjusting to the demands of medical school. Although the benefits of peer mentoring are well documented, less is understood about how these relationships support development for both mentees and mentors. This study explored how peer mentoring relationships were formed, maintained and contributed to students' academic, social and professional growth.

**Methods:**

The study was conducted in 2021–2022 at the Southeast Asian branch campus of a UK medical school, where second‐year students mentored first‐year students through a nine‐month voluntary peer mentoring programme. Mentors completed structured training and met regularly with their mentees across the academic year. A concurrent nested mixed‐method design was used, incorporating modified surveys and semi‐structured focus groups. Reflexive thematic analysis was guided by the Community of Practice (CoP) framework.

**Findings:**

Twenty‐three mentors and mentees participated in focus groups, whereas 64 mentees and 12 mentors completed the survey. Participants described a strong sense of purpose, rooted in a desire to help others and give back through peer mentoring. Mentoring fostered connection, belonging and professional identity through collaboration, emotional support and open communication. Participants gained practical benefits such as academic assistance, smoother social integration and strengthened interpersonal skills. Challenges included disengagement, cultural differences and mismatched expectations.

**Conclusion:**

This study highlights how the CoP framework fosters effective mentoring relationships, where shared goals, mutual engagement and collective practices promote academic, social and professional development. Educators should apply this framework to build inclusive, supportive communities, with future research exploring long‐term outcomes and cultural responsiveness.

## 1 Background

First‐year students often face numerous challenges in adapting to the academic, social and emotional demands of medical school [1, 2]. Peer mentoring programmes, where more experienced students guide newer students, have been shown to bring significant academic and psychosocial benefits, supporting students' acculturation to university and fostering their overall success [3, 4]. These programmes are beneficial in easing stress, improving teamwork, enhancing self‐confidence and developing both personal and professional qualities [5, 6]. Mentees benefit from mutual relationships with mentors and from acquiring essential academic and professional skills [7]. This is particularly important for students who typically experience differential attainment, such as those from underrepresented or minority backgrounds [8]. For mentors, the experience enhances personal growth, leadership and communication skills [9, 10], while also providing rewarding emotional and professional experiences [11, 12]. Compared with faculty mentors, near‐peer mentors, defined as peers one or two academic years ahead who offer guidance based on recently acquired knowledge, tend to be more approachable for mentees and foster more frequent interactions [13, 14].

Despite the wealth of literature describing the positive outcomes of peer mentoring for first‐year medical students and their mentors, there remains a gap in understanding how these relationships are shaped and sustained. Existing studies on peer mentoring often focus on outcomes rather than the mechanisms driving these relationships [15]. For instance, although Antonji et al. [16] identified significant psychosocial and academic benefits, these studies largely overlook how mentors and mentees develop a shared domain of interest and navigate mutual challenges. Furthermore, most studies focus on faculty‐led mentorship, limiting insight into how near‐peer mentoring facilitates these outcomes [1, 17]. Although Cho and Lee [18] provided us with a more comprehensive view of the factors contributing to mentoring success, including emotional support and relational bonding, there remains a gap in the literature to explain why the peer mentoring programme works and how these dynamics unfold.

### 1.1 Theoretical Framework

Sociocultural learning theories emphasise that learning occurs through participation in collaborative social environments [15]. Within peer mentoring, mentors create spaces that encourage dialogue and feedback that maximise mentees' learning potential [19]. A key concept that encapsulates this process is Community of Practice (CoP), which refers to groups whose members share a domain of interest and, through sustained interaction, develop a repertoire of shared practices [20]. The Association for Medical Education in Europe (AMEE) has proposed that mentors can foster learning by encouraging mentees to engage in CoPs, enabling them to move from legitimate peripheral participation towards fuller membership and professional identity [21, 22]. Despite the growing use of peer mentoring in medical education, limited research has examined how CoPs shape the development and sustainability of these relationships, particularly in near‐peer contexts [15]. Leveraging the three core elements of the CoP framework provides a useful lens for examining how peer mentoring relationships develop and support learning: The *domain* highlights the shared goals that unite mentors and mentees; the *community* element draws attention to the relationships and social structure that foster sustained engagement; the *practice* dimension focuses on the shared resources exchanged that drive learning and identity formation. Together, these components offer a useful lens for examining how near‐peer mentoring supports learning and integration into the medical school environment.

### 1.2 Study Aim and Research Question

This study aimed to explore the mechanisms by which peer mentoring relationships were formed, sustained and contributed to the personal, academic and professional development of medical students. Using the CoP framework, the study investigated how mentors and mentees engaged, collaborated and created shared practices that facilitated learning and integration within the medical school environment. The overarching goal was to gain insight into the relational dynamics that drove the success of peer mentoring programmes. Our specific research question was:

How did medical students facilitate meaningful mentor–mentee relationships and create supportive learning environments to foster academic and professional development?

Understanding the research phenomenon provides deeper insights into how mentoring relationships were cultivated and sustained, offering practical recommendations for strengthening peer mentoring programmes.

## 2 Methods

### 2.1 Study Design

This study was conducted at Newcastle University Medicine Malaysia, the international branch campus of Newcastle University Medical School in Johor, Malaysia. The phenomenon of interest in this research was students' perceptions of who participated in the mentor–mentee programme, which was examined within an interpretivist paradigm. We adopted a concurrent nested mixed‐method study design where the qualitative methodology was the dominant method, with the smaller quantitative data set embedded within the qualitative outcomes, playing a supplementary role in providing a better understanding of the phenomenon [23]. We analysed both data sets separately and integrated them during the interpretation and reporting phase. The quantitative and qualitative results were triangulated and corroborated for interrelationships to increase the breadth and depth of understanding. We adopted a theory‐informing inductive data analysis study design where we employed the CoP theory [20] as a lens through which we interpreted our data.

### 2.2 Contextual Background

**FIGURE 1:**
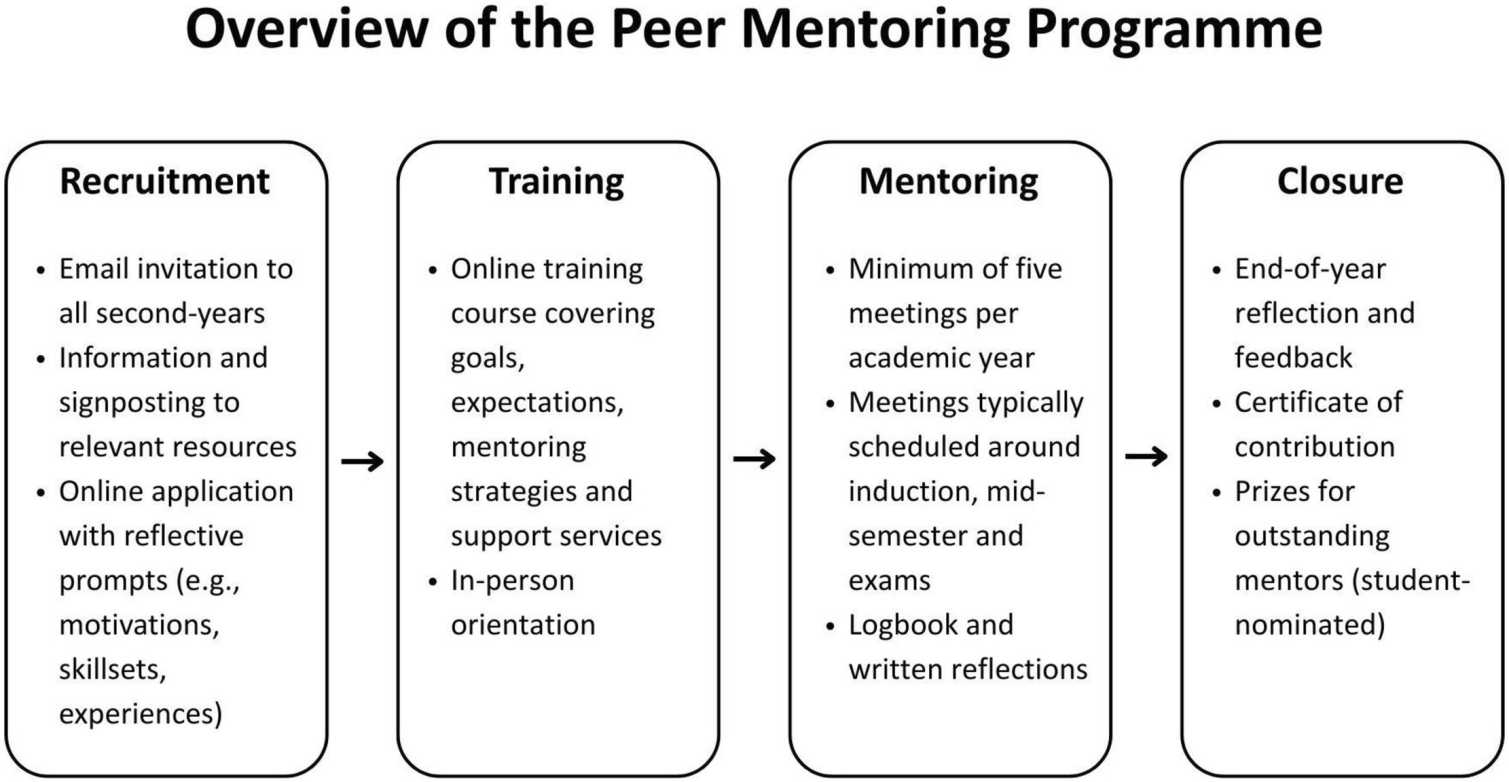
Overview of the peer mentoring programme, outlining key stages: recruitment, training, mentoring activities and programme closure.

Our research focused on the peer mentoring programme conducted during the 2021–2022 academic year. The 9‐month programme involved 36 second‐year medical student volunteers (mentors) who were paired with 108 first‐year medical students (mentees). To recruit mentors, programme coordinators sent a campus‐wide email inviting all second‐year medical students to participate. Interested students completed an online survey that included demographic questions and short reflective prompts about their motivations and relevant skills. Selected mentors completed an online training course on Canvas, which included a quiz and covered programme goals, mentoring strategies, session ideas, institutional expectations and available support services. This was followed by an in‐person orientation led by the programme coordinators. Mentors were required to hold at least five meetings with their mentees across the academic year, typically timed around induction, mid‐term and exam periods. Sessions were informal and flexible, commonly covering academic life, well‐being, university services and social integration. Mentors also maintained a logbook, submitted reflections and engaged with coordinators via a dedicated Microsoft Teams channel. Participation was voluntary and unpaid, though certificates and prizes were awarded in recognition of their contributions (see Figure 1 for an overview of the programme).

### 2.3 Data Collection

Semi‐structured focus group discussions (FGDs) were chosen to enable theoretical framing while facilitating the inductive and interpretative nature of qualitative data collection. JL and GK designed the interview schedule based on CoP theory to ensure grounding in previously published literature and alignment with existing theoretical constructs. The questions focused on three key areas: the shared domain of interest and the drive for proficiency; collaborative activities that foster learning relationships; and the collective development of shared language, resources, concepts, experiences and tools through interactions. We invite all mentors and mentees (*n* = 144) to participate in FGDs. To collect data representative of students' experiences across different stages of their medical education, we also purposively recruited senior medical students who are former mentors to participate in FGDs via snowball sampling. Those who expressed interest were provided with a participant information sheet and given written, voluntary consent before the interviews. To minimise potential power dynamics, JL and GK conducted all FGDs.

To supplement our qualitative data, we performed literature searches, group consultations and feedback surveys with mentors and mentees to identify themes about student perceptions of the programme. The themes were used in modifying the questionnaire by Hryciw et al. [24] through the refinement, replacement and development of additional items to make them more applicable to medical students. Modification of the questionnaire yielded nine items for mentors and 18 items for mentees, which explored their motivation and skills development. Cronbach's alpha coefficient for the mentor questionnaire was 0.87 and the mentee questionnaire was 0.96, which is satisfactory. All mentors and mentees were invited via institutional emails to fill out the questionnaire on the JISC online survey platform (see the Supporting Information for questionnaire).

### 2.4 Data Analysis

We adopted Braun and Clarke's six‐phase reflexive thematic analysis [25]. All FGDs were transcribed verbatim by JL and GK and kept reflective notes in a separate document. JL and GK then independently conducted open coding line‐by‐line on the transcripts with NVivo 14 (QSR International Pty Ltd. Version 14, 2023), meeting weekly to compare their work, discuss discrepancies and refine a coding scheme that captured recurring themes and patterns. In the second phase of analysis, VV and NN independently re‐analysed a portion of the qualitative data, aligning with the process of investigator triangulation to enhance the trustworthiness of the data. All members provided input into the iterative development of codes and final themes through regular project meetings to encourage peer critique and to reach an agreement. We believe our sample provided sufficient information power given our focused aim of exploring medical students' perspectives and experiences with the mentorship programme, which is further supported by our highly specific sample (i.e., mentors and mentees), the application of CoP theory in analysing our data and the in‐depth discussion during our interviews [26]. Quantitative survey data were analysed descriptively using frequencies and percentages to summarise responses to each Likert‐scale item. These results were grouped according to the three domains of the CoP framework. The quantitative findings were then compared with themes emerging from the qualitative data to identify areas of alignment and divergence, thereby enhancing interpretation through methodological triangulation.

### 2.5 Reflexivity

Our research team included two non‐clinical lecturers (VV and NN) and two clinical‐year medical students (GK, Year 3; JL, Final Year), all originally from the Global South with transnational educational experiences in the United Kingdom. VV has led the NUMed peer mentorship programme since 2016, and GK and JL have participated in the programme as both mentees and mentors. JL and GK reflected on how their own mentoring experiences could shape interpretation, whereas VV and NN considered how their roles as programme coordinators might predispose them to focus on success indicators such as university transition and academic performance. We acknowledged that such perspectives might limit awareness of concerns more salient to students, so we encouraged each team member to challenge assumptions around student motivations and experiences during regular team discussions. We also remained sensitive to the unique cultural context: NUMed applies a UK curriculum within a Southeast Asian setting, where mentoring norms tend to be more hierarchical. This dual cultural lens required us to consider how local expectations and Western pedagogical models may have shaped participant experiences and our interpretations.

### 2.6 Ethical Approval

Ethical approval for this study was granted by the Faculty of Medical Sciences, Newcastle University Ethics Committee.

## 3 Findings

### 3.1 Demographics

Five FGDs were conducted with nine mentees and 14 mentors, lasting between 48 and 63 min. Participants were divided according to their nationality and year groups: FGD1 (Year 1 local mentees), FGD2 (Year 1 international mentees), FGD3 (Year 2 mentors), FGD4 (Year 3 former mentors) and FGD5 (Year 4 former mentors). A total of 64 mentees and 12 mentors participated in the survey, yielding response rates of 58% and 30%, respectively. The majority were female (59% of mentees and 58% of mentors) and local (67% of mentees and 58% of mentors) and had two to four meetings throughout the mentorship period (52%). Most FGDs lasted 1 h or less (53%) and took place via virtual platforms (70%). The demographics of the participants in the FGDs and questionnaire are presented in Table 1.

**TABLE 1:** Characteristics of the participants.

Criteria		Respondents	
		*n*	%
Focus group discussions			
Mentees (*n* = 9)			
Gender	Female	7	59
	Male	2	41
Nationality	Local	6	67
	International	3	33
Year	First	9	100
Mentors (*n* = 14)			
Gender	Female	9	64
	Male	5	36
Nationality	Local	7	50
	International	7	50
Year	Second	4	30
	Third	5	35
	Fourth	5	35
Questionnaire			
Mentees (*n* = 64)			
Gender	Female	38	78
	Male	26	22
Nationality	Local	43	67
	International	21	33
Year	First	64	100
First language	Arabic	1	2
	Chinese	27	42
	English	17	27
	Indian	15	23
	Malay	4	6
Number of meetings	0–1	21	33
	2–4	33	52
	5 or more	10	15
Duration of meetings	1 h or below	34	53
	More than 1 h	7	11
	No limit	23	36
Format of meetings	Online	45	70
	In‐person	4	7
	Both	15	23
Mentors (*n* = 12)			
Gender	Female	7	58
	Male	5	42
Nationality	Local	7	58
	International	5	42
Year	Second	12	100
First language	Indian	4	33
	English	3	25
	Chinese	3	25
	Arabic	1	8
	Malay	1	8
Pre‐university qualification	A‐levels	5	42
	Foundation	4	33
	Others	3	25

### 3.2 Domain

To address our research question, we organise the findings into three sections based on the CoP framework: domain, community and practice. The dominant shared domain of interest that binds mentors together is their desire to give back to the learning community:

I really like talking to and helping people, like I volunteer a lot. I just like talking to all these junior medical students and sharing my experience, I think it's a good opportunity to meet new people, share your experiences, and make the medical school a better experience for them. (Year 2 mentor, female, local student)

All mentors described a shared purpose of wanting to help the next batch of students using their own experience and skills because they felt a personal obligation to give back, inspired by their own positive mentoring experiences. This motivation was evident in the way mentors leveraged their academic feedback:

I signed up for peer mentoring to share my assignment feedback as a guide to the next batch of year 1s, because I hope they don't make the same mistake that we have made. (Year 2 mentor, female, local student)

Some mentors found that shared cultural experiences helped them connect more easily:

I was able to help international students because we had similar experiences, and it was easy to relate and connect. (Year 2 mentor, female, international student)

Mentees, in turn, were inspired by their mentors and aspired to become mentors themselves in future years. Many described how they felt compelled to ‘pay it forward’ and create a similarly supportive environment for incoming students:

Personally, what really motivated me to be a peer mentor was my peer mentor. When I first joined the medical school, I was a bit confused. I was not sure how I would get along with things, I thought I might need support along the way, and I really appreciated the fact that I had a peer mentor. She was amazing in the way she handled everything. So when I had queries she was very tactful, and she created an expectation of how a mentor should be. I wanted to implement those lessons with my mentees. (Year 2 mentor, male, international student)

The questionnaire provided quantitative results that supported our qualitative findings (see Table 2). All mentors (100%) stated that they became mentors because of a desire to ‘make a difference’ and because they ‘enjoy helping others’. Additionally, 92% of mentors cited meeting new people as a key motivator for taking on the role. These findings demonstrate how a shared purpose grounded in reciprocity and the desire to give back forms the basis of the mentoring domain.

**TABLE 2:** Questionnaire results.

Statements		Likert score*					
		*Strongly disagree*	*Disagree*	*Slightly disagree*	*Slightly agree*	*Agree*	*Strongly agree*
Mentor (*n* = 12) (%)							
Domain							
1	To make a difference to others	0	0	0	1 (8.3)	6 (50)	5 (8.3)
2	Enjoy helping others	0	0	0	0 (0.0)	4 (8.3)	8 (8.3)
3	To meet new people	0	0	1 (8.3)	0 (0.0)	6 (50)	5 (8.3)
Community							
4	Feel more connected to the university	0	0	0	2 (16.7)	6 (50.0)	4 (33.3)
5	Developed a positive relationship with my mentee	0	0	0	1 (8.3)	7 (58.3)	4 (33.3)
Practice							
6	Developed problem‐solving skills	1 (8.3)	0	0	4 (33.3)	4 (33.3)	3 (25.0)
7	Developed confidence	0	0	0	1 (8.3)	6 (50.0)	5 (41.7)
8	Developed a high self‐esteem	0	0	0	3 (25.0)	6 (50.0)	3 (25.0)
9	Developed communication skills	0	0	0	0 (0.0)	8 (66.7)	4 (33.3)
Mentee (*n* = 64) (%)							
Community							
1	Allowed me to meet new people	0 (0.0)	7 (10.9)	5 (7.8)	21 (32.8)	19 (29.7)	12 (18.8)
2	Felt I have a peer group	1 (1.6)	7 (10.9)	4 (6.4)	16 (25.0)	21 (32.8)	15 (23.4)
3	Felt comfortable talking to my mentor	1 (1.6)	2 (3.2)	2 (3.2)	12 (18.8)	24 (37.4)	23 (35.8)
4	Felt comfortable in the university	3 (4.7)	3 (4.7)	0 (0.0)	15 (23.4)	25 (39.1)	18 (28.1)
5	Developed trust to my mentor	1 (1.6)	3 (4.8)	4 (6.4)	10 (15.5)	24 (37.4)	22 (34.3)
Practice							
6	Developed problem‐solving skills	10 (15.6)	6 (9.4)	2 (3.1)	21 (32.8)	20 (31.2)	5 (7.8)
7	Developed confidence	2 (3.1)	3 (4.7)	2 (3.1)	18 (28.1)	26 (40.6)	13 (20.3)
8	Developed a high self‐esteem	13 (20.3)	10 (15.6)	3 (4.7)	15 (23.4)	17 (26.6)	6 (9.4)
9	Developed communication skills	5 (7.8)	5 (7.8)	0 (0.0)	15 (23.4)	26 (40.6)	13 (20.3)
10	Developed organisational skills	6 (9.4)	7 (10.9)	1 (1.6)	16 (25)	28 (43.8)	6 (9.4)
11	Developed time management skills	6 (9.4)	8 (12.5)	2 (3.1)	23 (35.9)	20 (31.2)	5 (7.8)
12	Developed coping skills	7 (10.9)	5 (7.8)	1 (1.6)	20 (31.1)	24 (37.5)	7 (10.9)
13	Improved social interactions	6 (9.4)	6 (9.4)	1 (1.6)	17 (26.6)	21 (32.8)	13 (20.8)
14	Improved knowledge in MBBS subject	2 (3.1)	3 (4.7)	5 (7.8)	13 (20.3)	30 (46.9)	11 (17.2)
15	Helped with ways to tackle studies	3 (4.7)	4 (6.3)	2 (3.1)	13 (20.3)	26 (40.6)	16 (25)
16	Increased awareness of academic resources	3 (4.7)	2 (3.1)	2 (3.1)	18 (20.3)	26 (40.6)	12 (18.8)
17	Reduce stress level	6 (9.4)	3 (4.7)	3 (4.7)	21 (32.8)	24 (37.5)	7 (10.9)
18	Increased awareness of the pastoral services	4 (6.3)	9 (14.0)	3 (4.7)	22 (34.4)	20 (31.2)	6 (9.4)

*Note:* Asterisk describes the Likert scale used. The participants rated the extent to which they agreed with statements using a 6‐point Likert scale (1–6).

### 3.3 Community

Mentors, mentees and their broader peer groups are actively engaged in collaborative, supportive, meaningful activities that strengthen relationships and create a supportive learning environment. Many mentors went above and beyond their formal duties to engage with students who were not their direct mentees and support them:

There are a lot of other mentors who go beyond and help out. Before my first semester exam, there was one second‐year student, she's not my mentor, but she's a very nice person and listed down all the resources that you could get for your examination, and we talked about things that are non‐academic related as well, she was like my fairy godmother. Not only me, but she actually helps a lot of my friends. All of us went to her when we needed help. (Year 1 mentee, female, local student)

Mentors encourage open dialogue and accessibility using technology, such as WhatsApp groups, for mentees to text them or call them directly. Maintaining a casual relationship was a common strategy among mentors to maximise engagement. Mentors aim to be a reliable source of support and strive to be someone their mentees can turn to when needed:

We made a WhatsApp group so the mentees can reach out to us in whatever way they want, they can call us individually, or they can just text us directly. I am very open with my mentees, and they are very open and honest with me, and whenever they needed help, they could just approach me. (Year 3 mentor, male, international student)

Mentees often expressed how their mentors provided not only academic but also emotional and social support:

When I had COVID, my mentor helped me and told me what to do, and at times when I was burnt out, I had someone to rely on. I joined a lot of clubs, started having more friends and developing a social life, I personally wouldn't have done it if my mentor wasn't there. (Year 1 mentee, male, international student)

For international mentees, mentors with similar linguistic and cultural backgrounds helped ease their adjustment to the local environment.

I'm an international student, and so was my peer mentor, both of us did not speak the local language, she's Indian and I am Pakistani. So, we both kind of speak the same language, so she gave me advice on how to fit in and learn the language. (Year 1 mentee, male, international student)

The questionnaire revealed that the peer mentoring programme fostered a strong sense of connection and trust between participants (see Table 2). All mentors (100%) agreed that the programme helped them feel more connected to the University, and they were able to develop positive relationships with their mentees. Similarly, 92% of mentees felt comfortable communicating with their mentors, 87% developed trust in their mentors and 81% felt that the programme provided them with a supportive peer group.

Despite these positive experiences, some mentors faced challenges with disengaged mentees. Demographic differences, particularly nationality, also presented obstacles, with international students citing being unable to help local students due to unfamiliarity, and vice versa. Finally, mentors found it challenging to manage expectations, particularly around academic success:

I felt really stressed when my mentees told me the advice I gave them did not help them get the marks they wanted. (Year 2 mentor, male, international student)

### 3.4 Practice

As part of the peer mentoring community, both mentors and mentees developed a shared set of resources and valuable skills that formed the foundation of their collective practice. Mentors reported growth in transferable and interpersonal skills. Additionally, they highlighted how the programme reinforced their professional identity as future doctors.

I developed leadership skills as the programme gave me a sense of responsibility when it mandated us to do meetings and give advice to mentees. I also developed time management skills as I had to squeeze in time between classes, assignments, and my mentees’ deadlines, as well as exams, and with the time zone difference, which was quite a challenge. As future doctors, we'd be teaching juniors so I think the whole peer mentoring aspect of it familiarises you with the medical profession in the long run. (Year 4 mentor, female, local student)

Quantitative findings further corroborated the findings related to skill development, with 91.7% of mentors reporting improvements in communication skills and confidence, whereas 83.3% agreed they had enhanced their problem‐solving abilities (see Table 2).

Mentees similarly described improvements in study strategies, stress management and interpersonal interactions. Furthermore, their transition into the MBBS curriculum was supported by mentors who shared academic advice and personal experiences:

For year one especially when we first come to medical school, we're very lost in terms of revision methods and types of assignments we're not really used to, so this is all not familiar, so the whole idea of familiarising yourself with a new system with the peer mentor is really good, because it can be very intimidating to ask like lecturers, or even your academic mentor. (Year 1 mentee, female, local student)

Survey findings echoed these themes, with 65.6% of mentees reporting enhanced MBBS subject knowledge and support in study strategies. Varying levels of improvement were also noted across key transferrable skills, including communication, time management and coping abilities (see Table 2).

## 4 Discussion

### 4.1 Summary of Key Findings

We explored the experiences of first‐ and second‐year medical students as mentors and mentees through the lens of CoP. We found that mentors and mentees alike developed a sense of shared purpose, with mentors motivated by a desire to help others through leveraging their past experiences, feedback and connections, whereas mentees are inspired to become future mentors. Their mutual engagement was apparent through the strong relationships formed, where mentors engaged mentees through collaborations, open communication, going above and beyond and fostering cross‐mentoring relationships. This mentorship process fostered a sense of belonging for mentees as they gradually transitioned from newcomers to legitimate members and developed their professional identity within the academic and professional community. Lastly, their shared practice included valuable academic resources, problem‐solving strategies, psychological support and social communication techniques, which strengthened their communication, leadership and professional skills and, in turn, aided their career and professional development.

This mentorship process fostered a sense of belonging for mentees as they gradually transitioned from newcomers to legitimate members.

**FIGURE 2:**
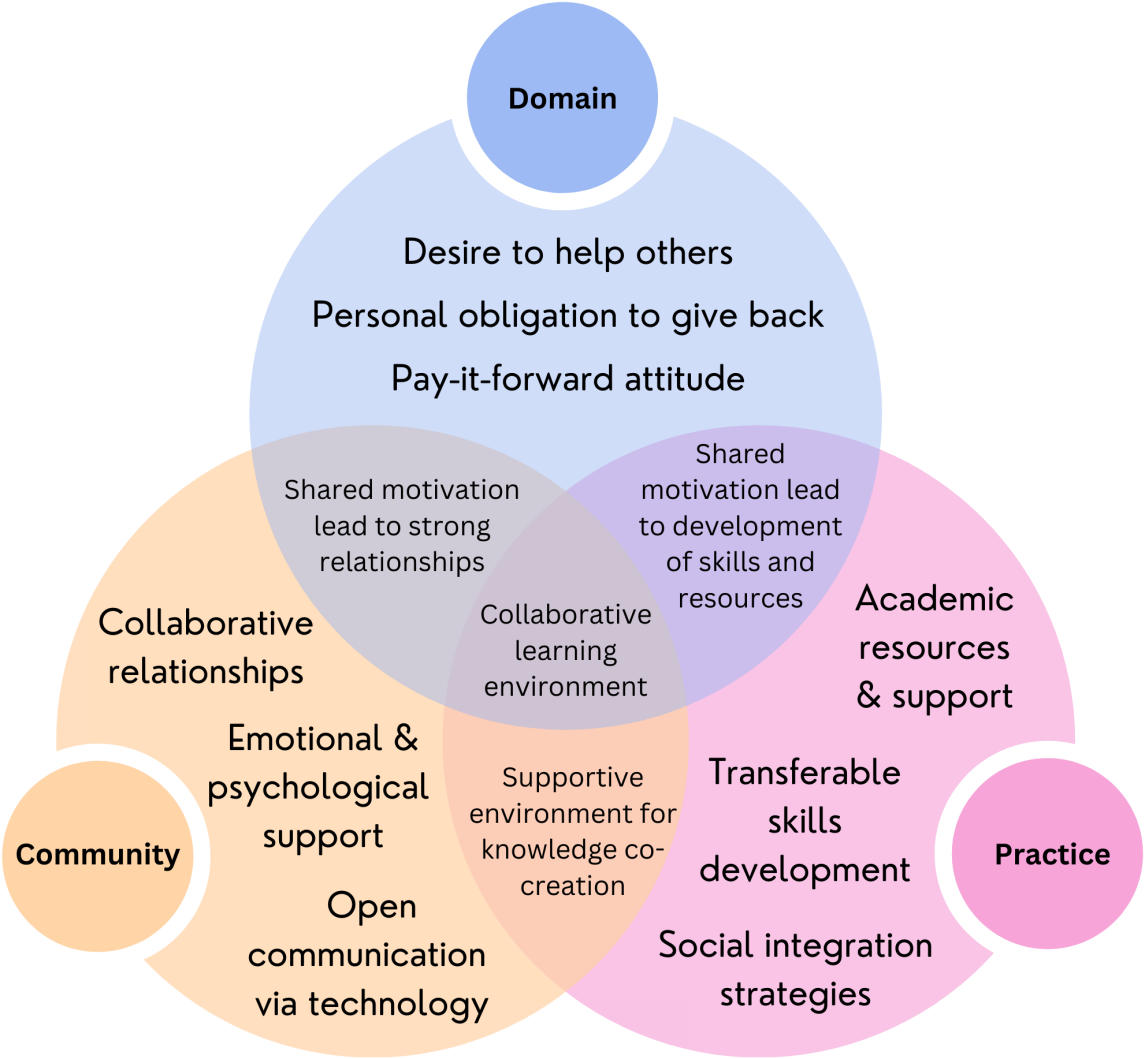
A three‐circle Venn diagram illustrating the interaction of the three core components of CoPs within the peer mentoring programme.

Fundamentally, the CoP theory helped illuminate how the interactions between mentors and mentees created a collaborative learning environment, which we illustrated using a three‐circle Venn diagram to show how the core components of CoPs interconnect to achieve this outcome in the peer mentoring programme (see Figure 2). Each circle represents a CoP element, and their overlaps demonstrate interactions between these dimensions: Shared motivation strengthens relationships within the community (domain + community), the community provides a supportive environment for knowledge co‐creation (community + practice), and shared motivation drives the development of skills and resources (domain + practice). At the center, the overlap of all three elements represents a collaborative learning environment where mentors and mentees mutually benefit. Mentees gain social, academic and psychological support, whereas mentors enhance leadership, communication and professional skills. The programme fosters mutual growth through shared purpose, meaningful engagement and the reinforcement of personal and professional development. It also sustains the community by cultivating a ‘pay it forward’ culture, as mentees aspire to become mentors, perpetuating the cycle of mentorship and creating a self‐sustaining community of learners.

The programme fosters mutual growth through shared purpose, meaningful engagement and the reinforcement of personal and professional development.

### 4.2 Comparison With Existing Literature

Our findings are consistent with previous research on the dual benefits of peer mentoring in both academic and professional development [5, 18]. However, this study extends the existing literature by illustrating how these benefits are facilitated through the framework of CoP [20]. We found that mentees exhibited positive behavioural changes through active participation in the academic community, aligning with level three of Kirkpatrick's 4‐level framework for evaluating training outcomes, which includes: (1) Reaction (participants' satisfaction), (2) Learning (knowledge acquisition), (3) Behaviour (knowledge application) and (4) Results (real‐world performance). A close mentor–mentee relationship enabled mentees to access the community's shared repertoire of knowledge and skills, echoing Wenger's view where mentees, as newcomers, journey from legitimate peripheral participation to gradually transition into full members of the community. Our findings resonate with the literature [16] where mentors' positive behaviours, such as approachability and supportiveness, correlate with positive academic and psychosocial outcomes of the mentees and foster collegiality and a sense of belonging [13].

Within a CoP, learning extends beyond knowledge acquisition to the construction of a professional identity. Through participation in shared tasks, assuming increasing responsibility and immersion in the community's practices, mentors strengthen their professional identities as future doctors [27]. Despite these benefits, challenges in sustaining mentor–mentee relationships were evident, particularly when cultural and nationality differences were present. This finding is in line with Marshall et al. [11], who noted that such differences can sometimes hinder connection. This suggests the need for more culturally tailored mentoring approaches or validated matching processes that allow for greater flexibility in mentor selection [28]. Additionally, mentors often find it difficult to balance their mentoring responsibilities with academic commitments, which underscores the importance of providing them with adequate training and resources [15]. Such support would help them manage their workload while maintaining boundaries and staying accessible to mentees [6, 28].

Within a CoP, learning extends beyond knowledge acquisition to the construction of a professional identity.

### 4.3 Implications for Educators and Researchers

By encouraging a joint purpose, supportive community and a shared repertoire of resources, educators can leverage this CoP‐inspired mentorship framework to create collaborative environments where first‐ and second‐year medical students (mentees and mentors) experience enhanced support, integration and development. The challenges identified highlight the importance of developing targeted training programmes that equip mentors with the skills to effectively manage their time, set appropriate boundaries and handle cultural differences with sensitivity. Training should also include strategies for fostering inclusive mentoring relationships that address the diverse needs of mentees, particularly those from varied cultural or international backgrounds. This study suggests that mentorship programmes can serve as powerful tools for promoting leadership development and shaping the professional identities of future professionals. Embedding mentorship into undergraduate medical programmes as a formalised component can help institutions cultivate a sense of community and professional growth among students.

Mentorship programmes can serve as powerful tools for promoting leadership development and shaping the professional identities.

Future studies should investigate the long‐term impacts of sustained involvement in CoP‐based mentorship programmes on both mentors and mentees through longitudinal observational studies. Our study highlights the importance of cultural sensitivity in mentoring relationships, particularly when mentees and mentors come from diverse national or cultural backgrounds. Researchers should further explore how tailored mentoring approaches that account for cultural differences can enhance the effectiveness of peer mentoring programmes. By investigating the specific challenges and opportunities faced by international or culturally diverse student populations, future studies can identify strategies for creating more inclusive and supportive mentoring environments. Lastly, future research could explore how workload pressures impact the effectiveness of peer mentoring programmes and the well‐being of mentors, as the understanding of how to mitigate burnout among mentors while maintaining strong mentor–mentee relationships could inform the design of more sustainable and supportive mentoring structures.

### 4.4 Methodological Strengths and Challenges

This study has several methodological strengths and limitations that should be considered when interpreting the findings. One of the key strengths is the strong alignment between our chosen theoretical framework, research question, data collection and analysis, which enhances the credibility of our findings, whereas the use of detailed quotes improves the confirmability of our qualitative data [29]. The nested mixed‐methods design allows for triangulation of qualitative and quantitative findings, and our rigorous reflexive practices, such as maintaining reflective journals, further improve qualitative rigour. Additionally, our diverse sample in terms of nationality, ethnicity and first languages contributes to a broader understanding of peer mentoring across different cultural and linguistic backgrounds. Notwithstanding, our study faces several challenges. A key limitation is the low survey response rate from peer mentors (30%), which introduces the potential for response bias. It is possible that those who responded had particularly positive experiences with the programme, which may skew the quantitative findings. Although the quantitative component served a supplementary role to our primary qualitative findings [30], this limitation still warrants caution in interpretation. Another limitation is that our findings are based on a single undergraduate medical programme delivered in Malaysia, aligned with the guidelines of the General Medical Council and the Malaysian Medical Council. These specific contextual factors, specifically the curriculum structure, institutional practices and cultural settings, may limit the generalisability of our results to other settings, such as postgraduate medicine, non‐medical mentorship programmes or medical degrees delivered outside Malaysia and the United Kingdom.

## 5 Conclusion

In conclusion, this study illuminated the mechanisms that drive successful mentoring relationships, as framed by CoP theory, demonstrating how shared domains of interest, mutual engagement and collective practices contribute to a supportive and collaborative learning environment. Educational institutions should leverage the CoP framework to build inclusive, supportive communities that empower students to thrive. Future research should focus on exploring the long‐term impact of the CoP‐based peer mentorship programme and exploring strategies to foster cultural inclusivity and diversity within the community.

Educational institutions should leverage the CoP framework to build inclusive, supportive communities that empower students to thrive.

## Author Contributions

**Jun Jie Lim:** methodology, investigation, writing – original draft, formal analysis, project administration, writing – review and editing. **Vivian Andaya Verbo:** conceptualisation, supervision, funding acquisition, methodology, investigation, formal analysis, project administration, writing – review and editing. **Gunjan Khandelwal:** investigation, formal analysis, writing – review and editing. **Nadine Hayudini Nograles:** conceptualisation, supervision, funding acquisition, methodology, investigation, formal analysis, project administration, writing – review and editing.

## Ethics Statement

Ethical approval was obtained from the Faculty of Medical Sciences, Newcastle University Ethics Committee.

## Conflicts of Interest

The authors declare no conflicts of interest.

## Supporting information


**Data S1:** Mentor questionnaire.

## Data Availability

The data that support the findings of this study are available from the corresponding author upon reasonable request.
