# Supplementary material for: Peer Mentoring as a Community of Practice in Medical Education
Source: Clin Teach. 2025 Nov 5;22(6):e70238. doi: 10.1111/tct.70238 (PMC12588802; doi:10.1111/tct.70238)
Supplement: Supplementary file 1 — Data S1: Mentor questionnaire. [file TCT-22-e70238-s001.docx]

**Supplementary Material**

**Supplementary Material 1: Mentor questionnaire**

**Section 1: Demographic Information**

1. **What is your gender?**
   ☐ Male
   ☐ Female
2. **What is your nationality?**
   ☐ Local
   ☐ International
3. **What is your first language?**
4. **What are your pre-university qualifications?**

**Section 2: Motivation to become a mentor**

Please rate the following motivations for you becoming a mentor on a scale from 1 to 6, where:
1 = Strongly Disagree, 2 = Disagree, 3 = Slightly Disagree, 4 = Slightly Agree, 5 = Agree, 6 = Strongly Agree

| **Motivation** | **1** | **2** | **3** | **4** | **5** | **6** |
| --- | --- | --- | --- | --- | --- | --- |
| 1. Meet new people | ☐ | ☐ | ☐ | ☐ | ☐ | ☐ |
| 2. Make a difference to others | ☐ | ☐ | ☐ | ☐ | ☐ | ☐ |
| 3. Enjoy helping others | ☐ | ☐ | ☐ | ☐ | ☐ | ☐ |

**Section 3: Skills development**

Please rate the following academic, personal and social skills you have developed through the programme on a scale from 1 to 6, where:
1 = Strongly Disagree, 2 = Disagree, 3 = Slightly Disagree, 4 = Slightly Agree, 5 = Agree, 6 = Strongly Agree

| **Skills** | **1** | **2** | **3** | **4** | **5** | **6** |
| --- | --- | --- | --- | --- | --- | --- |
| 4. Improved my problem-solving skills | ☐ | ☐ | ☐ | ☐ | ☐ | ☐ |
| 5. Develop my confidence | ☐ | ☐ | ☐ | ☐ | ☐ | ☐ |
| 6. Develop a high self-esteem | ☐ | ☐ | ☐ | ☐ | ☐ | ☐ |
| 7. Develop my communication skills | ☐ | ☐ | ☐ | ☐ | ☐ | ☐ |
| 8. Feel more connected to the university | ☐ | ☐ | ☐ | ☐ | ☐ | ☐ |
| 9. Develop a positive relationship with my mentee(s) | ☐ | ☐ | ☐ | ☐ | ☐ | ☐ |

**Supplementary Material 2: Mentee questionnaire**

**Section 1: Demographic Information**

1. **What is your gender?**
   ☐ Male
   ☐ Female
2. **What is your nationality?**
   ☐ Local
   ☐ International
3. **What is your first language?**
4. **How many mentoring sessions have you attended in the first semester?**

☐ 0-1
☐ 2-4

☐ 5 or more

1. **What is the frequency of those meetings?**

☐ At least once per week

☐ At least once per month
☐ At least once per case

☐ Less than at least once per case

1. **What is the duration of each meeting?**

☐ 1 hour or below
☐ More than 1 hour

☐ No limit

1. **How was those meetings conducted?**

☐ Online
☐ In-person

☐ Both

**Section 2: Impact of Mentor Meetings on Mentee Development**

Please rate the extent to which meeting with your mentor as a mentee has impacted the following skills and areas of development on a scale from 1 to 6, where:
1 = Strongly Disagree, 2 = Disagree, 3 = Slightly Disagree, 4 = Slightly Agree, 5 = Agree, 6 = Strongly Agree

**2.1 Academic Skills**

| **Skills** | **1** | **2** | **3** | **4** | **5** | **6** |
| --- | --- | --- | --- | --- | --- | --- |
| 1. Helped my confidence in my studies | ☐ | ☐ | ☐ | ☐ | ☐ | ☐ |
| 2. Helped my knowledge in my current MBBS subjects | ☐ | ☐ | ☐ | ☐ | ☐ | ☐ |
| 3. Helped my organisational skills | ☐ | ☐ | ☐ | ☐ | ☐ | ☐ |
| 4. Helped my problem-solving skills | ☐ | ☐ | ☐ | ☐ | ☐ | ☐ |
| 5. Helped my time management skills | ☐ | ☐ | ☐ | ☐ | ☐ | ☐ |
| 6. Given me ways to tackle my studies | ☐ | ☐ | ☐ | ☐ | ☐ | ☐ |
| 7. Increased my awareness of academic resources | ☐ | ☐ | ☐ | ☐ | ☐ | ☐ |

**2.2 Emotional and Personal Development**

| **Skills** | **1** | **2** | **3** | **4** | **5** | **6** |
| --- | --- | --- | --- | --- | --- | --- |
| 8. Helped develop my coping skills | ☐ | ☐ | ☐ | ☐ | ☐ | ☐ |
| 9. Helped reduce my stress level | ☐ | ☐ | ☐ | ☐ | ☐ | ☐ |
| 10. Helped improve my self-esteem | ☐ | ☐ | ☐ | ☐ | ☐ | ☐ |
| 11. Increased my awareness of the pastoral care services (e.g., counselling services) | ☐ | ☐ | ☐ | ☐ | ☐ | ☐ |

**2.3 Social and Peer Engagement**

| **Skills** | **1** | **2** | **3** | **4** | **5** | **6** |
| --- | --- | --- | --- | --- | --- | --- |
| 12. Allowed me to meet new people | ☐ | ☐ | ☐ | ☐ | ☐ | ☐ |
| 13. Enhanced my social communication skills | ☐ | ☐ | ☐ | ☐ | ☐ | ☐ |
| 14. Increased my confidence in social interactions | ☐ | ☐ | ☐ | ☐ | ☐ | ☐ |
| 15. Helped me feel comfortable in the university environment | ☐ | ☐ | ☐ | ☐ | ☐ | ☐ |
| 16. Helped me feel that I have a peer group at university | ☐ | ☐ | ☐ | ☐ | ☐ | ☐ |
| 17. Helped me feel comfortable talking to my mentor | ☐ | ☐ | ☐ | ☐ | ☐ | ☐ |
| 18. Developed my trust in my mentor | ☐ | ☐ | ☐ | ☐ | ☐ | ☐ |
